# Supplementary figures and images for: Secreted Reporter Assay Enables Quantitative and Longitudinal Monitoring of Neuronal Activity
Source: eNeuro. 2021 Sep 29;8(5):ENEURO.0518-20.2021. doi: 10.1523/ENEURO.0518-20.2021 (PMC8489021; doi:10.1523/ENEURO.0518-20.2021)

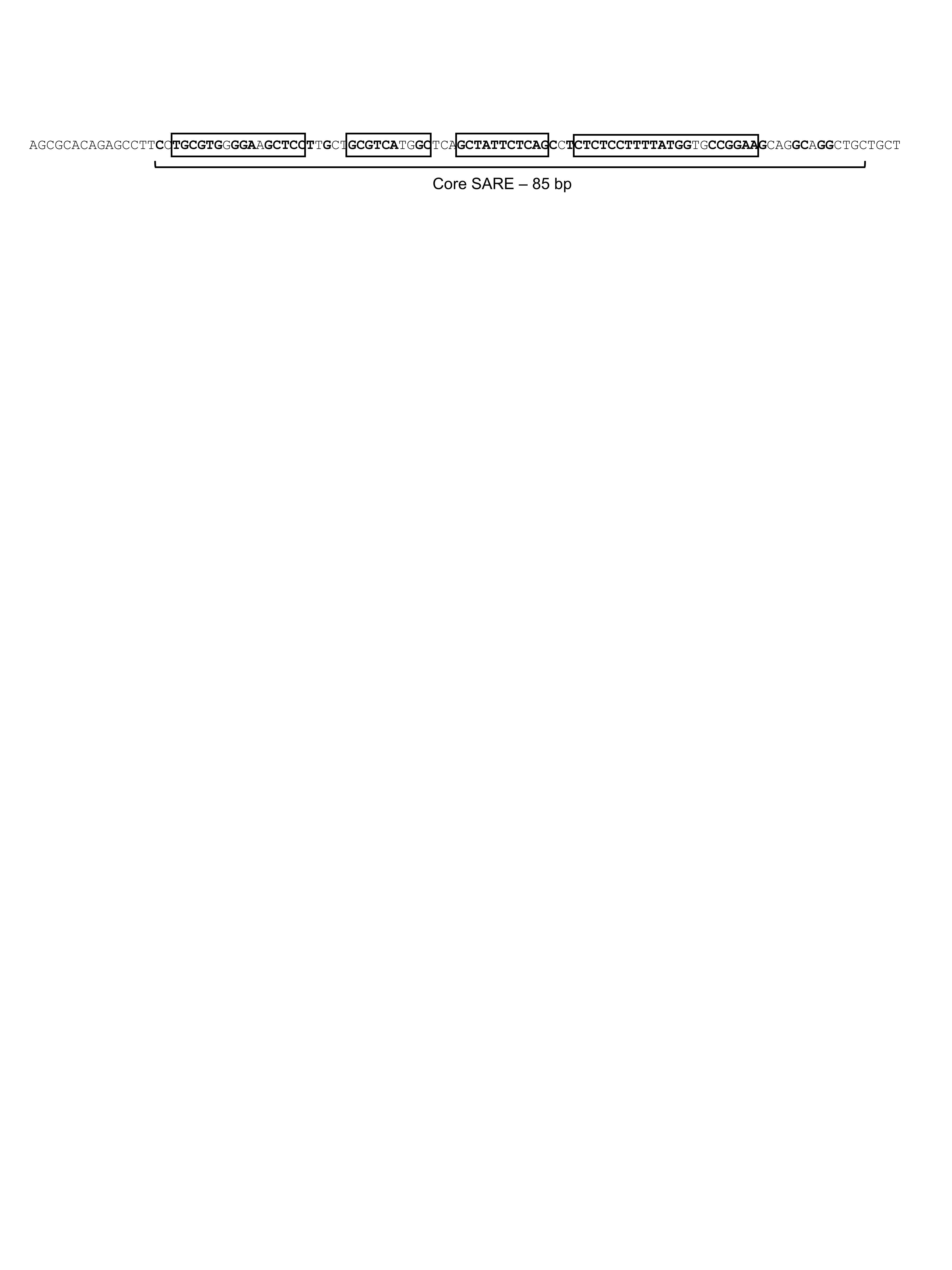

Supplement: Extended Data Figure 1-1 — The cSARE sequence. A, The full SARE is listed and the region that was used as cSARE is annotated. Boxes indicate conserved transcription factor binding sites that were used to determine this region and that may mediate SARE function. Bold characters indicate the conserved sequence between mouse and human. Download Figure 1-1, TIF file. [file enu-eN-MNT-0518-20-s03.tif]
